# Care delay during the COVID-19 pandemic in Germany – a cross-sectional online survey in the NAKO study

**DOI:** 10.1186/s12889-026-27202-w

**Published:** 2026-04-01

**Authors:** Janka Massag, Theresa Herrmann, Laura R. Pfrommer, Amand Führer, Klaus Berger, Hermann Brenner, Karina Karolina De Santis, Karin Halina Greiser, Volker Harth, André Karch, Thomas Keil, Muhammad Nasir Khan Khattak, Carolina J. Klett-Tammen, Lilian Krist, Niels O. Kristiansen, Benedikt Lampl, Michael Leitzmann, Wolfgang Lieb, Ute Mons, Ilais Moreno Velásquez, Alexandra Nieters, Nadia Obi, Cara Övermöhle, Anette Peters, Tobias Pischon, Börge Schmidt, Matthias B. Schulze, Kerstin Wirkner, Hajo Zeeb, Rafael Mikolajczyk

**Affiliations:** 1 Institute for Medical Epidemiology, Biometrics and Informatics (IMEBI), Interdisciplinary Centre for Health Sciences, Medical Faculty of the Martin Luther University Halle-Wittenberg, Universitätsmedizin Halle - Universitätsklinikum Halle (Saale), Magdeburger Straße 8, 06112 Halle (Saale), Germany; 2 Institute of Epidemiology and Social Medicine, University of Münster, Münster, Germany; 3 German Cancer Research Center (DKFZ), Heidelberg, Germany; 4 Leibniz Institute for Prevention Research and Epidemiology - BIPS, Bremen, Germany; 5 Institute of Occupational and Maritime Medicine (ZfAM), University Medical Center Hamburg-Eppendorf, Hamburg, Germany; 6 Institute of Social Medicine, Epidemiology and Health Economics, Charité - Universitätsmedizin Berlin, Berlin, Germany; 7 Institute of Clinical Epidemiology and Biometry, University of Würzburg, Würzburg, Germany; 8 State Institute of Health I, Bavarian Health and Food Safety Authority, Erlangen, Germany; 9 Department of Study of Health in Pomerania (SHIP)/ Clinical-Epidemiological Research (KEF), Institute for Community Medicine, University Medicine Greifswald, Greifswald, Germany; 10 Department of Epidemiology, Helmholtz Centre for Infection Research, Braunschweig, Germany; 11 Institute of Molecular Biology (IMB), Mainz, Germany; 12 Regensburg Department of Public Health, Regensburg, Germany; 13 Department of Epidemiology and Preventive Medicine, University of Regensburg, Regensburg, Germany; 14 Institute of Epidemiology, Kiel University, Kiel, Germany; 15 Max-Delbrück‐Center for Molecular Medicine in the Helmholtz Association (MDC), Molecular Epidemiology Research Group, Berlin, Germany; 16 Centre for Chronic Immunodeficiency, University Medical Center Freiburg, Freiburg, Germany; 17 Institute of Epidemiology, Helmholtz Zentrum München, German Research Center for Environmental Health (GmbH), Neuherberg, Germany; 18 Institute for Medical Informatics, Biometry and Epidemiology, University Hospital of Essen, University of Duisburg-Essen, Essen, Germany; 19 Department of Molecular Epidemiology, German Institute of Human Nutrition Potsdam-Rehbruecke, Nuthetal, Germany; 20 Institute of Nutritional Science, University of Potsdam, Nuthetal, Germany; 21 Leipzig Research Centre for Civilization Diseases (LIFE), Leipzig University, Leipzig, Germany

**Keywords:** COVID-19 Pandemic, Health Services Accessibility, Delayed Diagnosis, Germany, NAKO, Survey

## Abstract

**Background:**

During the COVID-19 pandemic, non-COVID-19 related healthcare utilization declined in Germany, resulting in care delay, including delays and cancellations of routine, chronic, and even acute care. The aim of this study was to investigate factors (i.e. regional differences and participant characteristics) associated with care delay during the pandemic in Germany using a cross-sectional survey.

**Methods:**

In October 2022, a total of 117,466 participants from the German National Cohort (NAKO) study completed an online questionnaire on pandemic-related topics, including care delay during the COVID-19 pandemic. Regional differences and participant characteristics associated with care delay were assessed using (multilevel) logistic regression.

**Results:**

One third of participants reported having experienced care delay. Care delay did not differ across the 13 federal states or 32 districts in Germany for which sufficient data were available. In the medical practice setting, care delay was nearly equally provider- and patient-related and was reported mostly for routine check-ups. In the hospital setting, care delay was predominantly provider-related and reported for newly occurring conditions. The odds for care delay were higher in females vs. males (odds ratio (OR): 1.30; 95% confidence interval (CI): 1.27–1.34), and in participants with vs. without chronic conditions (e.g. mental disorders, OR: 1.41, 95%CI: 1.36–1.46 or cardiovascular diseases, OR: 1.20 95%CI: 1.16–1.24) and decreased with age (e.g. 70 + vs. 50–59 years, OR: 0.59, 95%CI: 0.57–0.62).

**Conclusion:**

Care delay during the COVID-19 pandemic depended on participant characteristics including age, sex, and preexisting chronic conditions but not on regional (i.e. state and district-level) differences in Germany.

**Supplementary Information:**

The online version contains supplementary material available at 10.1186/s12889-026-27202-w.

## Introduction

During the COVID-19 pandemic, a decrease in healthcare utilization for non-COVID-19 conditions was observed worldwide and contributed to wide-ranging care delay, including delays and cancellations of routine, chronic, and even acute care [1]. Between 20% and 40% of individuals seeking medical care in various countries and healthcare settings reported delays in care or not receiving the medical attention they needed [2–8].

Care delay was common particularly for routine care and chronic conditions [9]. However, also acute conditions like myocardial infarction were affected [1]. Patient characteristics associated with an increased risk of care delay included sex (higher in females), high education [3], lower socioeconomic status [9], lack of insurance, chronic illness, and belonging to racial or ethnic minorities [2]. In contrast, older age (65 + years) appeared to be a protective factor against care delay [2, 3].

The most commonly reported reasons for care delay include providers canceling appointments [9] and patients postponing or canceling appointments due to financial difficulties [4] or fear of SARS-CoV-2 infection [5]. However, evidence remains inconclusive whether provider or patient-related factors mainly contributed to the care delay [6, 10, 11].

Most studies on care delays during the pandemic focused on distinct medical specialties or specific conditions and not on healthcare in general, which could be one explanation for the inconsistent findings among the studies [1]. Furthermore, most studies were conducted in the United States [1], where the absence of universal healthcare limits the generalizability of results to European settings. However, even within Europe, studies showed notable differences in care delays during the pandemic across countries, including Germany, France, Italy, the United Kingdom, and Spain [12]. Potential regional disparities, such as those related to healthcare availability in rural or socioeconomically-disadvantaged regions, within those countries have not been investigated to date.

Germany presents a unique case for assessing regional disparity within a European country due to its historical division into East and West Germany, which operated under two entirely different economic and political systems and reunified in 1990. Socioeconomic inequalities between East and West Germany persist to this day [13, 14]. However, disparities are also growing at smaller regional levels, for example within and between the 16 federal states and 401 districts in Germany. Those regional inequalities reflect the socioeconomic and infrastructural differences in healthcare indicators, such as the density of general practitioners at regional level within Germany [15] and are greater than in other European countries [13].

This study aims to examine factors (i.e. regional differences and participant characteristics) associated with care delay during the COVID-19 pandemic in Germany. The specific objectives of this study were: (1) to describe the frequency and type of care delay during the COVID-19 pandemic in Germany, (2) to describe the perceived consequences of any care delay, and (3) to investigate the factors (i.e. regional differences and participant characteristics) associated with any care delay and with care delay stratified by type.

## Methods

### Study design and reporting

This study uses a cross-sectional design and is based on an online-survey as part of the population-based German National Cohort (NAKO Gesundheitsstudie) study. The reporting adheres to the Strengthening the Reporting of Observational Studies in Epidemiology (STROBE) guideline (Additional file 1) [16].

### Data source

The study uses data from participants of the NAKO study, a prospective, longitudinal epidemiological study that investigates risk factors of common diseases and geographic and socio-economic disparities in health status of the general population in Germany. Potential participants were selected through age- and sex-stratified random sampling from population registries. The NAKO aimed to recruit 10,000 individuals per 10-year age group between 20 and 39 years and 26,667 individuals per 10-year age group between 40 and 69 years for both males and females. Between 2014 and 2019, around 205,000 adults aged 19 to 74 years were recruited in 18 study centers across Germany, covering 13 federal states and 49 districts. Baseline data were collected on-site through in-person interviews, self-reported questionnaires, biological samples and from population registries. More information on the study design of the NAKO is provided elsewhere [17].

### Ethical considerations

Ethical approval for the NAKO study was obtained from all local ethics committees of the 18 study centers. The implementation of optional additional study modules was planned in the study design. The study was conducted in accordance with the Declaration of Helsinki with the exception that the study was not registered before the recruitment started, and all participants provided written informed consent.

### Participants

The inclusion criteria for this study were participation in the NAKO study, availability of baseline sociodemographic data, valid email address, consent to be contacted by email and age above 20 years.

### Procedure

For this study, all NAKO participants with valid email addresses (*n* = 150,722) were invited to participate in an online survey between October and December 2022. The questionnaire was administered in LimeSurvey that could be accessed through an individual link sent to participants via email. Participants could navigate backwards in the questionnaire to alter their answers and pause and return to the survey at any time before the final submission.

### Instrument (Questionnaire)

The data were collected via a self-developed questionnaire in German. The questionnaire included multiple topic sections related to the pandemic. The section used for this study consisted of 14 items covering care delay. Although the questionnaire was not formally validated in a separate empirical study, it was developed based on instruments used in previous pandemic-related research and refined through expert consensus across all participating study centers, supporting its content and face validity. The 14 items had either 3–5 closed answer options (e.g. yes, no, some) or required a single numerical answer (e.g. the number of times medical appointments were cancelled; Additional file 2).

Participants were asked if they had experienced any care delay and if so, if such care delay was patient or provider-related (i.e., the appointment was canceled or postponed by the participant or the provider, respectively) and if it occurred in a medical practice or hospital setting. In the German health care system, most regular care is taking place in medical practices (general practitioners or specialists), whereas in hospitals surgeries or more complex diagnostic or therapeutic procedures are conducted. The answers were recoded into three variables: (1) any care delay (yes or no), (2) care delay provider-related (yes or no), (3) care delay patient-related (yes or no). Participants with care delay were also asked follow-up questions for each scenario (e.g. about the number and the type of cancelled appointments and if they were partially or completely caught up). Participants were also asked whether they perceived that any care delay negatively impacted their health, thereby assessing their general subjective perception.

The baseline data included information on participant date of birth, sex, education, household net income, medical history, and district of the participant place of residence. The information on date of birth, sex and place of residence was retrieved from the population registries used for recruitment. The place of residence was available as the official administrative key for the German counties (Kreisschlüssel). This five-digit number gives information about the federal state and district. This key was also matched with data from the Federal Institute for Research on Building, Urban Affairs and Spatial Development to classify the residence regions into urban and rural areas. Education was determined based on the highest school and vocational qualification reported by the participants. To determine the medical history the participants were asked if they ever received any diagnosis of cardiovascular disease, metabolic disease and psychiatric disorders and were given multiple choices of pre-defined diagnosis for each group. Additionally, they were asked if they ever received a diagnosis of cancer and had to report the cancer type in an open question. Participants’ health insurance status (i.e. statutory, private, or other) was introduced as a variable only later in the study. Therefore, since insurance status was not available for 51.6% (60,564 of 117,466) of participants in the current analysis, this variable was not included in the analysis.

### Statistical analysis

All statistical analysis was conducted in R Version 4.5.0.

Relative and absolute frequencies were computed for categorical variables and mean and standard deviation (SD) or median and inter-quartile range (IQR) for continuous variables.

To examine regional differences on district and federal state level, multilevel logistic regression models were constructed according to the procedure described by Sommet and Morselli [18]. A total of six multilevel models were constructed, each using one of the following dependent variables: any care delay, provider-related delay, and patient-related delay. As level-2 grouping variables, either the state of residence (*n* = 13) or the district of residence (included if the district had at least 100 participants, *n* = 32) was used. In a first step, an empty model containing just the dependent variable and the level-2 variable as a random effect was evaluated using the *glmer* function from the *lme4* package [19]. To assess the degree of clustering at the level-2 grouping variable, the intraclass correlation coefficient (ICC) was calculated based on the variance components of the empty model, using the following formula:

$$\:ICC=\:\frac{Var\left({u}_{0j}\right)}{Var\left({u}_{0j}\right)+\frac{{\pi\:}^{2}}{3}}$$

In case of the ICC being different from zero, district and federal state would be included as random effects in the model. The individual variables sex, age, education, income and chronic conditions and regional variables East/West/Berlin and urban/rural were included as fixed effects. As a sensitivity analysis we additionally ran the models only for participants with preexisting chronic conditions, who might have distinct care needs.

In a second step, we tested for potential interactions between regional and individual characteristics. This included interactions between East/West/Berlin and sex, East/West/Berlin and age, as well as the three-way interaction East/West/Berlin × sex × age. Model selection was based on a hierarchical approach using the Akaike Information Criterion (AIC) and Bayesian Information Criterion (BIC), with the best-fitting model chosen according to the lowest BIC value [20]. All regression models are based on a complete case analysis.

## Results

### Response

Out of the 150,722 invited NAKO participants, 117,466 (77.9%) completed the online questionnaire. Characteristics of non-responders are shown in the Additional file 3 (Table S1). Due to the large sample size, the differences between responders and non-responders were not tested for statistical significance. However, a visual inspection of trends showed that non-responders tended to be younger than 49 years and male. Due to missing data (5.8% for income, 5.1% for education and below 1% for all other variables) the final sample included in the regression analysis was *n* = 106.191 participants with complete data (70.5% of the invited participants).

### Participant characteristics

Among the 117,466 participants, sex was evenly distributed (51.1% female vs. 48.9% male) (Table 1). The majority of participants reported at least one chronic condition diagnosed by a medical doctor (77.3%), with the highest prevalence of metabolic diseases (39.9%), followed by cardiovascular diseases (30.5%). A majority had a high level of education (62.3%), lived in the former West Germany (66.2%) and in urban areas (79.6%), were 50 years and older at the time of data collection (67.9%). Among participants with available data (*n* = 56,902), 45,508 (80.0%) had statutory health insurance, 10,869 (19.1%) had private insurance, and 525 (0.9%) were classified as ‘other.

**Table 1:** Participants‘ characteristics by any care delay during the pandemic (*n* = 117,466)

Characteristics^a^	Total	Any care delay	No care delay
	*n* = 117,466 (100%)	*n* = 35,157 (29.9%)	*n* = 82,308 (70.1%)
**Age group**^b^ (n (%))			
20–29	3,693 (3.1)	1,037 (2.9)	2,656 (3.2)
30–39	14,382 (12.2)	4,730 (13.5)	9,652 (11.7)
40–49	19,580 (16.7)	6,721 (19.1)	12,859 (15.6)
50–59	36,473 (31.0)	11,372 (32.3)	25,101 (30.5)
60–69	27,714 (23.6)	7,708 (21.9)	20,005 (24.3)
70+	15,624 (13.3)	3,589 (10.2)	12,035 (14.6)
**Sex**^c^ (n (%))			
Male	57,460 (48.9)	15,144 (43.1)	42,315 (51.4)
Female	60,006 (51.1)	20,013 (56.9)	39,993 (48.6)
**Education**^d^ (n (%))			
High	71,567 (62.3)	21,087 (61.3)	50,480 (62.7)
Middle/Low	43,294 (37.7)	13,301 (38.7)	29,993 (37.1)
**Equivalized net household income in €**^e^ (Median (IQR))	2,150 (1,297)	2,115 (1.250)	2,262 (1.417)
**Federal state**^f^ (n (%))			
Baden-Wurttemberg	13,879 (11.8)	4,147 (11.8)	9,732 (11.8)
Bavaria	17,326 (14.8)	4,951 (14.1)	12,375 (15.0)
Berlin	16,276 (13.9)	5,117 (14.6)	11,159 (13.6)
Brandenburg	4,060 (3.5)	1,296 (3.7)	2,764 (3.4)
Bremen	6,984 (5.9)	2,168 (6.2)	4,815 (5.9)
Hamburg	6,538 (5.6)	2,046 (5.8)	4,492 (5.5)
Mecklenburg-Western Pomerania	8,466 (7.2)	2,615 (7.4)	5,851 (7.1)
Lower Saxony	4,713 (4.0)	1,346 (3.8)	3,367 (4.1)
North Rhine-Westphalia	17,331 (14.8)	4,959 (14.1)	12,372 (15.0)
Saarland	5,978 (5.1)	1,802 (5.1)	4,176 (5.1)
Saxony	5,618 (4.8)	1,730 (4.9)	3,888 (4.7)
Saxony-Anhalt	5,251 (4.5)	1,558 (4.4)	3,693 (4.5)
Schleswig-Holstein	5,040 (4.3)	1,421 (4.0)	3,619 (4.4)
**East/West/Berlin**^g^ (n (%))			
East	23,395 (19.9)	7,199 (20.5)	16,196 (19.7)
West	77,789 (66.2)	22,840 (65.0)	54,948 (66.8)
Berlin	16,276 (13.9)	5,114 (14.6)	11,159 (13.6)
**Urban/Rural**^h^ (n (%))			
Urban	93,494 (79.6)	27,876 (79.3)	65,617 (79.7)
Rural	23,966 (20.4)	7,280 (20.7)	16,686 (20.3)
**Any cardiovascular disease**^i^ (n(%))			
Yes	35,515 (30.5)	10,937 (31.4)	24,577 (30.1)
No	81,034 (69.5)	23,925 (68.6)	57,109 (69.9)
**Any cancer**^j^ (n(%))			
Yes	7,746 (6.6)	2,428 (6.9)	5,318 (6.5)
No	109,437 (93.4)	32,629 (93.1)	76,807 (93.5)
**Any metabolic disease**^k^ (n(%))			
Yes	46,506 (39.9)	14,909 (42.8)	31,596 (38.7)
No	69,956 (60.1)	19,928 (57.2)	50,028 (61.3)
**Any mental disorder**^l^ (n(%))			
Yes	19,420 (16.6)	7,302 (20.9)	12,118 (14.8)
No	97,329 (83.4)	27,628 (79.1)	69,701 (85.2)

^a^ All variables collected as part of the NAKO baseline data (2014–2019); ^b^ Age was grouped in 10-year brackets based on the NAKO sampling strategy; ^c^ Sex coded as a binary variable in population registries used for the NAKO recruitment; ^d^ Education levels according to the ISCED-97; ^e^ Net household income divided by households needs weight (1 for first adult household member + 0.5 for each further adult household member + 0.3 for every child under 14); ^f^ The NAKO includes participants from 13 out of the 16 German federal states; ^g^ Federal states of former East Germany: Brandenburg, Mecklenburg-Western Pomerania, Saxony and Saxony-Anhalt, federal states of the former West Germany: Baden-Wurttemberg, Bavaria, Bremen, Hamburg, Lower Saxony, North Rhine-Westphalia, Saarland and Schleswig-Holstein; Berlin was divided into East and West sector and thus is included as a third category; ^h^ According to the classification of the Federal Institute for Research on Building, Urban Affairs and Spatial Development; ^i^ Self-reported medical diagnosis of myocardial infarction, angina pectoris, cardiac insufficiency, cardiac arrhythmia, intermittent claudication or high blood pressure; ^j^ Self-reported medical diagnosis of cancer; ^k^ Self-reported medical diagnosis of diabetes, elevated blood lipids, gout or thyroid disease; ^l^ Self-reported medical diagnosis of depression or generalized anxiety disorder/panic disorder

### Care delay

#### Frequency of any care delay by participant characteristics

One third of participants (*n* = 35,157, 29.9%) reported having experienced any care delay during the pandemic (Table 1). Participants reporting care delay relative to those without care delay were more frequently female (56.9% vs. 48.6%), aged between 30 and 59 years (64.9% vs. 57.8%), with metabolic diseases (42.8% vs. 38.7%) and mental disorders (20.9% vs. 14.8%). The frequency of cardiovascular diseases (31.4% vs. 30.1%) and cancer (6.9% vs. 6.5%) was similar in those with vs. without care delay.

#### Type of care delay

Among participants who reported care delay (*n* = 35,156), half reported care delay in the medical practice setting. This was evenly attributed to provider-related (47.6%) and patient-related (46.0%) reasons (Table 2). Care delay was reported less frequently in the hospital settings with more frequent provider-related (11.5%) than patient-related (4.2%) delay.

**Table 2:** Type of care delay (*n* = 35,156 participants with any care delay)

	*n* (%) participants reporting respective type of care delay^a^
**Provider-related cancellation in medical practice setting**	16,719 (47.6)
Number of cancellations (Median (IQR))	2 (1)
Type of appointment cancelled (*n* = 16,719)	
Follow-up visit	5,407 (32.3)
Preventive	9,519 (56.9)
New condition	3,346 (20.0)
Other	3,078 (18.4)
Appointment(s) caught up (*n* = 16,463)	
All appointment(s)	12,586 (76.5)
Some appointments	2,684 (16.3)
No appointments	1,193(7.2)
**Patient-related cancellation in medical practice setting**	16,173 (46.0)
Number of cancellations (Median (IQR)	2 (1)
Type of appointment cancelled (*n* = 16,173)	
Follow-up visit	4,927 (30.5)
Preventive	10,056 (62.2)
New condition	2,044 (12.6)
Other	2,455 (15.2)
Appointment(s) caught up (*n* = 15,730)	
All appointment(s)	11,224 (71.4)
Some appointments	2,879 (18.3)
No appointments	1,627 (10.3)
**Provider-related cancellation in hospital setting**	4,052 (11.5)
Number of cancellations (Median (IQR)	1(1)
Type of appointment cancelled (*n* = 4,052)	
Follow-up visit	1,043 (25.7)
Preventive	372 (9.2)
New condition	1,738 (42.9)
Other	1,109 (27.4)
Appointment(s) caught up (*n* = 3,946)	
All appointment(s)	2,954 (74.9)
Some appointments	298 (7.6)
No appointments	694 (17.6)
**Patient-related cancellation in hospital setting**	1,490 (4.2)
Number of cancellations (Median (IQR))	1(1)
Type of appointment cancelled (*n* = 1,490)	
Follow-up visit	446 (29.9)
Preventive	211 (14.2)
New condition	404 (27.1)
Other	370 (24.8)
Appointment(s) caught up (*n* = 1,315)	
All appointment(s)	715 (54.4)
Some appointments	146 (11.1)
No appointments	454 (34.5)

^a^ Multiple responses were allowed; totals may exceed the number of participants

Care delay also differed by type of appointment. In the medical practice setting, provider-related and patient-related care delay was most frequent for preventive appointments (i.e. routine check-ups; 56.9% and 62.2%). In the hospital setting, most provider-related care delay was for newly occurring conditions (42.9%), whereas patient-related care delay was equally common for appointments for existing and newly occurring conditions (29.9% and 27.1%, respectively). In over 70% of cases, cancelled appointments in medical practice and hospital settings were caught up. However, only about half of the appointments cancelled by patients in the hospital setting were rescheduled.

#### Perceived negative effect of care delay on participant health

Of the participants who reported care delay and answered the question about the health impact of care delay (*n* = 29,074), 15.7% (*n* = 4,562) stated that they believed it had a negative effect on their health. This perception was higher among participants for whom not all cancelled appointments were caught up (19.0%; 3,756/19,783) relative to those for whom all cancelled appointments were caught up (7.6%; 628/8,533).

### Factors associated with care delay

#### Regional differences in care delay

For all the models that considered the different regional levels (district and federal state) and the different care delays (any, patient-related and provider-related), the ICC was ~ 0 (Table 3), indicating that there are no differences in care delay based on regional clustering. Therefore, we did not include district or federal state as random effects in the final models.

**Table 3:** Intraclass correlation coefficients for regional differences in any care delay and in care delay stratified by type

	Federal States	Districts
Any care delay	0.001	0.001
Patient-related care delay	0.002	0.001
Provider-related care delay	0.002	0.004

Multilevel logistic regression models were constructed in R using the glmer function from the lme4 package for the independent variables any care delay, patient-related care delay and provider-related care delay, respectively. Grouping (level-2) variables (federal state and district) were included as random effects. The ICC was calculated based on the variance components of the respective model, using the following formula: ICC= (Var(u_0j))/(Var(u_0j )+π^2/3)

#### Factors associated with any care delay

The details of the model selection criteria are shown in Additional file 3 (Tables S2 – S4). For all outcomes, the best model fit according to the BIC was the model with no interaction terms.

Care delay depended on participant characteristics, including age group, sex, chronic conditions, and income (Table 4). Care delay was more likely to be reported by participants who were younger than 50 years (i.e. between 30 and 49 years), female (vs. male), with chronic conditions (vs. without chronic conditions), especially mental disorders, and living in Berlin (vs. living in the former West Germany). At the same time, care delay was less likely to be reported by participants who were older than 59 years and with a higher equalized net household income (per 1,000€ increase). The associations changed only marginally when the analysis was restricted to participants with chronic conditions (Additional file 3, Table S5).

**Table 4:** Association between participant and regional characteristics and care delay (*n* = 106.191 with complete data)

	Any care delay	Patient-related care delay		Provider-related care delay
	Odds Ratio (95%-CI)			
**Age**				
20–29 years	0.95 (0.85–1.05)	0.79 (0.68–0.92)		0.75 (0.65–0.86)
30–39 years	1.18 (1.13–1.24)	1.16 (1.09–1.22)		0.98 (0.93–1.04)
40–49 years	1.19 (1.15–1.24)	1.25 (1.19–1.31)		1.12 (1.07–1.17)
50–59 years	1.00 (Reference)			
60–69 years	0.80 (0.77–0.83)	0.81 (0.77–0.85)		0.80 (0.76–0.84)
70 + years	0.59 (0.57–0.62)	0.58 (0.55–0.62)		0.54 (0.51–0.57)
**Sex**				
Male	1.00 (Reference)			
Female	1.30 (1.27–1.34)	1.66 (1.60–1.72)		1.20 (1.16–1.24)
**Education**				
High	1.00 (Reference)			
Middle/Low	0.97 (0.94–1.00)	0.86 (0.83–0.89)		0.97 (0.94–1.01)
**Equivalized net household income (per 1000 €)**	0.94 (0.93–0.95)	0.96 (0.95–0.98)		0.95 (0.93–0.96)
**Any cancer**				
No	1.00 (Reference)			
Yes	1.15 (1.09–1.21)	1.18 (1.10–1.26)		1.20 (1.13–1.28)
**Any cardiovascular disease**				
No	1.00 (Reference)			
Yes	1.20 (1.16–1.24)	1.22 (1.17–1.27)		1.24 (1.19–1.28)
**Any metabolic disease**				
No	1.00 (Reference)			
Yes	1.20 (1.16–1.23)	1.20 (1.15–1.24)		1.20 (1.15–1.24)
**Any psychiatric disorder**				
No	1.00 (Reference)			
Yes	1.41 (1.36–1.46)	1.42 (1.36–1.48)		1.40 (1.34–1.45)
**East/West/Berlin**				
West	1.00 (Reference)			
East	1.02 (0.98–1.06)	0.87 (0.82–0.91)		1.07 (1.02–1.13)
Berlin	1.10 (1.06–1.14)	1.10 (1.05–1.16)		1.15 (1.10–1.21)
**Urban/rural**				
Urban	1.00 (Reference)			
Rural	1.01 (0.98–1.05)	1.07 (1.02–1.13)		1.07 (1.02–1.13)

#### Factors associated with care delay stratified by type

A similar pattern of results was observed when care delay was stratified by type (i.e. provider- or patient-related; Table 4). In both cases, care delay was less likely to be reported by the youngest vs. older participants (i.e. aged 20–29 years vs. 50–59 years), while it was more likely to be reported by those living in rural vs. urban regions. In the case of patient-related care delay those with a low to middle (vs. high) education level and those living in the former East vs. West Germany were less likely to reported it. In contrast to patient-related care delay, participants living in the former East vs. West Germany reported more provider-related care delay.

## Discussion

One third of the sample reported care delay during the pandemic. Care delay was most common in the medical practice setting. In this setting, it was evenly split between patient- and provider-related delays and cancellations, and primarily affected preventive appointments. Care delay in the hospital setting was more commonly initiated by the provider and mostly affected appointments for newly occurring or existing conditions. Most appointments were eventually caught up and rescheduling was rarely perceived as having a negative impact on health. Patient characteristics most strongly associated with care delay were age, sex, and having a chronic condition, whereas sex had a stronger association with patient-related care delay compared to provider-related care delay. We found no differences in care delay on district and state level and only small differences for West/East/Berlin stratification.

### Lack of differences on state and district level in care delay during the pandemic

The only studies, we could find that looked at regional differences in care delay during the pandemic within a country were from the United States that also evaluated differences between rural and urban areas. Both studies did not find differences in the geographical distribution of care delay [7, 21]. This is in line with our results. Even though German districts and states were affected differently by the COVID-19 pandemic in terms of restrictions, infections and mortality and further differences in healthcare infrastructure [22], this did not seem to lead to regional differences in care delay. The smallest geographical unit, we could analyze in this study was the district level. Considerable variability in socioeconomic conditions and health indicators exists on smaller levels, even within German cities for example [23]. This is also reflected in the call for smaller-area health service research, with earlier studies showing variations in healthcare utilization across hospital service areas within a U.S. state [24] and greater variability in healthcare indicators between used healthcare facilities than for geographically defined region in Germany with a free choice of doctors [25]. This raises important questions regarding which regional or functional units are most appropriate for small-area health service research [26]. Further research could explore this question.

### Patient- and provider-related care delay

Regarding the question whether care delay during the pandemic was primarily provider- or patient-related, existing research is limited and findings remain inconsistent. A study with self-reports from older adults (60 + years) in Amsterdam found that up to early 2021, more cancellations were provider-initiated [6]. In contrast, a study conducted during the same period in southern Germany – focusing on medical practices and including a broader age range (25–88 years) – found that patient-related care delay was more frequent [10]. Our study, which covered a longer period (up to December 2022), showed no difference between provider- and patient-initiated care delay in medical practices. However, in the hospital setting we predominantly observed provider-initiated care delay.

Schepers et al. also observed that provider-related care delay was more common among specialists than general practitioners [10]. These findings highlight the need to account for both, the type of health care setting and the temporal context when analyzing care disruptions. They also underscore the importance of considering the diverse mechanisms behind care delays, which may vary by care setting and shift over the course of the pandemic. Our findings of most cancelled appointments being caught up during the pandemic underscore the importance of longer observation periods while investigating this topic, since only appointments that were not caught up seem to negatively impact health. Cancelled appointments could have been more of an issue in the first pandemic wave due to a lack of vaccination coverage, testing possibilities, and scarce knowledge on suitable prevention measures that improved in the later pandemic stages [6]. Thus, care delay may have changed across different pandemic phases and became less pronounced in the later stages [27].

### Factors associated with care delay

Regarding individual characteristics associated with care delay, the factors we identified largely align with the existing literature from the U.S. Identifying as female and living with a chronic condition (especially psychiatric conditions) were associated with increased care delay, while older age and lower education were associated with less care delay [2, 3]. In contrast to previous U.S.-based findings, we only saw a weak association between financial status and care delay. This difference may reflect structural disparities within health care systems, as healthcare access in the U.S. is more dependent on financial means due to the lack of universal health coverage, whereas in countries with universal healthcare, such as Germany, financial barriers may play a less prominent role [4, 9].

Additionally, similar associations were found, with minor variations depending on the type of care delay (patient- vs. provider-related). While education had no association with provider-related care delay, it was associated with patient-related care delay. Sex also had a stronger association with patient-related care delay compared to provider-related care delay. There were also different associations for the place of residence being in Berlin or other Eastern federal states or in states in the West of Germany. While patient-related care delay was less likely in Eastern Germany, provider-related care delay was more likely in Berlin. To our current knowledge, the differences in factors associated with patients- compared to provider-related care have not yet been studied. Our results indicate that there might be different rationales behind patient- and provider-related care delay during the COVID-19 pandemic. Shukla et al. proposed a framework on the individual decision to delay care during the pandemic based on an earlier framework on the general decision to delay care [28]. The general framework explains the decision to delay care as an interplay of the patient’s socio-cultural context, patient’s individual characteristics, aspects of the local health services and social network. Shukla et al. placed the focus in their framework on external and internal perceived risk and risk assessment and the trade-off between risk from COVID-19 and delaying care. This risk assessment might differ according to the characteristics we identified associated with delaying care, like sex, age, education and preexisting chronic conditions.

### Strengths and limitations

To our knowledge, this study was the first analysis of regional differences in care delay during the COVID-19 pandemic in Germany. This study could pinpoint towards methodological requirements of further investigations regarding regional differences for health care (delay) in Germany. A strength of the study was the high number of participants from different regions from Germany, which is unique due to the NAKO being the biggest epidemiological cohort in Germany. However, there were several limitations in this study. Despite the large sample size, there still might be a lack of regional representativeness in the data. The districts included in the study were non-randomly selected in proximity to the study centers, which are located, in most cases, in large cities. This selection approach may limit regional representativeness – especially in predominantly rural federal states like Saxony-Anhalt. In addition to restricted regional representativeness, there is also a limitation of participant representativeness within each region, which might have influenced our results. The NAKO study overall and the single study centers have an overrepresentation of participants with a high education level and older people, compared to the German general population [29, 30]. We cannot rule out possible bias by selection of the participants and regional areas. Thus, the lack of regional differences in care delay observed in this study may not be generalizable to all regions in Germany.

We acknowledge that the assessment of care delay in this study is limited by subjective perceptions in self-reported data. Furthermore, the participants had to choose between patient- and provider-related care delays although the decisions may have been made jointly. In any case, cancelling or postponing an appointment could have been accompanied by agreeing on a new appointment. Further research is needed to assess more detailed aspects of care delay. The analysis was also limited by a possibly blurred indicator for care delay, since we did not assess if participants (with our without care delay) had an appointment or healthcare need, nor how often appointments were not delayed. Our results of more care delay reported by females and people with chronic conditions are comparable with findings on general healthcare utilization [31]. The used indicator of care delay in this study might therefore also include information on the overall health care access and use patterns of the study participants, which could not be adjusted for. However, the question on the patient-related care delay was specifically asking for delay due to the pandemic and females were more likely to report patient- compared to provider-related care delay. Therefore, we can assume that at least part of the differences are due to pandemic-related factors.

## Conclusion

During the COVID-19 pandemic, both provider- and patient-related care delay were reported by a large sample of participants from a general population in 13 federal states in Germany. Care delay was associated with participant characteristics, including female sex, middle age, and existence of chronic conditions. In contrast, no meaningful regional differences in care delay were observed in this study. The latter finding is reassuring with respect to the sufficient access opportunities to healthcare under stress conditions during the pandemic in the 13 federal states. However, more research on access to healthcare is needed to cover less densely populated and socioeconomically disadvantaged regions in Germany. 

## Supplementary Information

Supplementary Material 1.

Supplementary Material 2.

Supplementary Material 3.

## Data Availability

The data that support the findings of this study are available from https://transfer.nako.de/transfer/index but restrictions apply to the availability of these data, which were used under license for the current study, and so are not publicly available. Data are however available from the authors upon reasonable request and with permission of NAKO e.V.
