# Supplementary material for: Care delay during the COVID-19 pandemic in Germany – a cross-sectional online survey in the NAKO study
Source: BMC Public Health. 2026 Apr 1;26:1172. doi: 10.1186/s12889-026-27202-w (PMC13063876; doi:10.1186/s12889-026-27202-w)
Supplement: Supplementary file 2 — Supplementary Material 2. [file 12889_2026_27202_MOESM2_ESM.docx]

**Additional file 2**

**Questionnaire in German (Original)**

**Fragebogen: Einschränkungen in der Medizinischen Versorgung während der COVID-19 Pandemie**

**F1**: Während der gesamten Pandemie gab es viele unterschiedliche Einschränkungen. In diesem Abschnitt möchten wir etwas über Einschränkungen in der medizinischen Versorgung erfahren. Haben Sie im gesamten Verlauf der Pandemie Einschränkungen Ihrer medizinischen Versorgung erfahren? Bitte alles Zutreffende ankreuzen.

1. Nein
2. Ja, ich hatte mindestens einen Termin in einer Arzt‐ /Zahnarztpraxis, der durch die Arzt‐ /Zahnarztpraxis abgesagt oder verschoben wurde
3. Ja, ich hatte mindestens einen Termin in einer Arzt‐ /Zahnarztpraxis, den ich selbst wegen der Pandemie abgesagt oder verschoben habe
4. Ja, ich hatte mindestens einen Termin in einem Krankenhaus, der durch das Krankenhaus abgesagt oder verschoben wurde
5. Ja, ich hatte mindestens einen Termin in einem Krankenhaus, den ich selbst wegen der Pandemie abgesagt oder verschoben habe

Termine die durch die Arzt-/Zahnarztpraxen abgesagt oder verschoben wurden

**F2** (Nur wenn F1=2): Wie häufig wurden Ihre Termine durch die Arzt‐ /Zahnarztpraxen abgesagt oder verschoben?

- |_|_| Mal

**F3** (Nur wenn F1=2): Um welche Art von Terminen handelte es sich dabei, die durch die Arzt‐/Zahnarztpraxen abgesagt oder verschoben wurden? Bitte alles Zutreffende ankreuzen.

1. Folgetermine bei Behandlung einer bekannten Erkrankung
2. Vorsorgetermine ohne zugrundeliegende bekannte Erkrankung
3. Behandlung einer neu aufgetretenen Erkrankung
4. Andere Termine

**F4** (Nur wenn F1=2): Wurden die durch die Arzt‐/Zahnarztpraxen abgesagt oder verschobenen Termine inzwischen nachgeholt?

1. Ja, alle Termine wurden nachgeholt
2. Einige ja, andere nicht
3. Nein, keiner der Termine wurde nachgeholt

Termine in Arzt-/Zahnarztpraxen, die durch Sie selbst abgesagt oder verschoben wurden

**F5** (Nur wenn F1=3): Wie häufig habe **Sie selbst** Termine in den Arzt‐ /Zahnarztpraxen abgesagt oder verschoben?

- |_|_| Mal

**F6** (Nur wenn F1=3): Um welche Art von Terminen handelte es sich dabei, die **Sie selbst** abgesagt oder verschoben haben? Bitte alles Zutreffende ankreuzen.

1. Folgetermine bei Behandlung einer bekannten Erkrankung
2. Vorsorgetermine ohne zugrundeliegende bekannte Erkrankung
3. Behandlung einer neu aufgetretenen Erkrankung
4. Andere Termine

**F7** (Nur wenn F1=3): Wurden die Termine in den Arzt‐/Zahnarztpraxen, die **Sie selbst** abgesagt oder verschobenen haben, inzwischen nachgeholt?

1. Ja, alle Termine wurden nachgeholt
2. Einige ja, andere nicht
3. Nein, keiner der Termine wurde nachgeholt

Krankenhausbehandlungen, die durch das Krankenhaus abgesagt oder verschoben wurden

**F8** (Nur wenn F1=4): Wie häufig wurde ein Termin im Krankenhaus durch das Krankenhaus abgesagt oder verschoben?

- |_|_| Mal

**F9** (Nur wenn F1=4): Um welche Art von Terminen handelte es sich, die durch das Krankenhaus abgesagt oder verschoben wurden? Bitte alles Zutreffende ankreuzen.

1. Folgetermine bei Behandlung einer bekannten Erkrankung
2. Vorsorgetermine ohne zugrundeliegende bekannte Erkrankung
3. Behandlung einer neu aufgetretenen Erkrankung
4. Andere Termine

**F10** (Nur wenn F1=4): Wurden die Termine, die durch das Krankenhaus abgesagt oder verschobenen wurden, inzwischen nachgeholt?

1. Ja, alle Termine wurden nachgeholt
2. Einige ja, andere nicht
3. Nein, keiner der Termine wurde nachgeholt

Krankenhausbehandlungen, die durch Sie selbst abgesagt oder verschoben wurden

**F11** (Nur wenn F1=5): Wie häufig haben **Sie selbst** Termine im Krankenhaus abgesagt oder verschoben?

- |_|_| Mal

**F12** (Nur wenn F1=5): Um welche Art von Terminen im Krankenhaus handelte es sich dabei, die **Sie selbst** abgesagt oder verschoben haben? Bitte alles Zutreffende ankreuzen.

1. Folgetermine bei Behandlung einer bekannten Erkrankung
2. Vorsorgetermine ohne zugrundeliegende bekannte Erkrankung
3. Behandlung einer neu aufgetretenen Erkrankung
4. Andere Termine

**F13** (Nur wenn F1=5): Wurden die durch **Sie selbst** verschobenen Termine im Krankenhaus inzwischen nachgeholt?

1. Ja, alle Termine wurden nachgeholt
2. Einige ja, andere nicht
3. Nein, keiner der Termine wurde nachgeholt

Auswirkungen des Ausfalls oder der Verschiebung der Behandlungstermine

**F14** (Nur wenn F1=2,3,4 oder 5): Denken Sie, dass sich der Ausfall oder die Verschiebung Ihrer

Behandlungstermine negativ auf Ihre Gesundheit ausgewirkt haben?

1. Ja
2. Eher ja
3. Eher nein
4. nein

**Questionnaire in English (Translation)**

**Questionnaire: Restrictions in medical care during the COVID-19 pandemic**

**Q1**: During the entire pandemic there were many different restrictions. In this part we want to learn something about restrictions in medical care. Did you experience restrictions in your medical care throughout the pandemic? Please select all that apply.

1. No
2. Yes, I had at least one appointment in a medical/dentist practice that was canceled or postponed by the medical/dentist practice.
3. Yes, I had at least one appointment in a medical/dentist practice that I myself canceled or postponed due to the pandemic.
4. Yes, I had at least one appointment in a hospital that was canceled or postponed by the hospital.
5. Yes, I had at least one appointment in a hospital that I myself canceled or postponed due to the pandemic.

Appointments canceled or postponed by medical/dentist practices

**Q2** (Only if Q1=2): How many times were your appointments canceled or postponed by the medical/dentist practice?

- |_|_| times

**Q3** (Only if Q1=2): Which types of appointments were canceled or postponed by the medical/dentist practice? Pleas select all that apply.

1. Follow-up appointment for a known condition
2. Preventive appointment without underlying known condition
3. Treatment of a new condition
4. Other appointments

**Q4** (Only if Q1=2): Were the appointments postponed by the medical/dentist practice caught up by now?

1. Yes, all appointments were caught up
2. Some yes, others no
3. No, none of the appointments were caught up

Appointments at medical/dentist practices that were cancelled or postponed by yourself

**Q5** (Only if Q1=3): How many times did **you yourself** cancel or postpone appointments at medical/dentist practices?

- |_|_| times

**Q6** (Only if Q1=3): Which types of appointments were canceled by **yourself**? Pleas select all that apply.

1. Follow-up appointment for a known condition
2. Preventive appointment without underlying known condition
3. Treatment of a new condition
4. Other appointments

**Q7** (Only if Q1=3): Were the appointments at the medical/dentist practices that **you yourself** postponed caught up by now?

1. Yes, all appointments were caught up

2. Some yes, others no

3. No, none of the appointments were caught up

Hospital treatments that were canceld or postponed by the hospital

**Q8** (Only if Q1=4): How many times was an appointment in a hospital canceled or postponed by a hospital?

- |_|_| times

**Q9** (Only if Q1=4): Which types of appointments were canceled or postponed by the hospital? Please select all that apply.

1. Follow-up appointment for a known condition
2. Preventive appointment without underlying known condition
3. Treatment of a new condition
4. Other appointments

**Q10** (Only if Q1=4): Were the appointments postponed by the hospital caught up by now?

1. Yes, all appointments were caught up
2. Some yes, others no
3. No, none of the appointments were caught up

Hospital treatments that were canceled or postponed by yourself

**Q11** (Only if Q1=5): How many times did **you yourself** cancel or postpone appointments at the hospital?

- |_|_| times

**Q12** (Only if Q1=5): Which types of appointments at the hospital were canceled or postponed by **yourself**? Please select all that apply.

1. Follow-up appointment for a known condition
2. Preventive appointment without underlying known condition
3. Treatment of a new condition
4. Other appointments

**Q13** (Only if Q1=5): Were the appointments at the hospital postponed by **you yourself** caught up by now?

1. Yes, all appointments were caught up

2. Some yes, others no

3. No, none oft he appointments were caught up

Impact oft he cancellation or postponment of appointments

**Q14** (Only if Q1=2,3,4 or 5): Do you think that the cancellation or postponement of your treatment appointments had a negative impact on your health?

1. Yes
2. Rather yes
3. Rather no
4. No
