# Supplementary material for: Care delay during the COVID-19 pandemic in Germany – a cross-sectional online survey in the NAKO study
Source: BMC Public Health. 2026 Apr 1;26:1172. doi: 10.1186/s12889-026-27202-w (PMC13063876; doi:10.1186/s12889-026-27202-w)
Supplement: Supplementary file 3 — Supplementary Material 3. [file 12889_2026_27202_MOESM3_ESM.docx]

**Additional file 3**

**Table S1: Characteristics of responders and non-responders to the online questionnaire**

|  | **Responder** | **Non-responder** | **Difference** |
| --- | --- | --- | --- |
| **n = 150,722** | 117,446 (77.9) | 33,257 (22.1) | Percentage points |
| **Age** **group** (n (%)) |  |  |  |
| 20-29 | 3,693 (3.14) | 1,859 (5.59) | 2.45 |
| 30-39 | 14,382 (12.24) | 5,793 (17.41) | 5.17 |
| 40-49 | 19,580 (16.67) | 6,162 (18.53) | 1.86 |
| 50-59 | 36,473 (31.05) | 9,336 (28.08) | 2.97 |
| 60+ | 43,338 (36.89) | 10,104 (30.38) | 6.51 |
| **Sex** (n (%)) |  |  |  |
| Male | 57,460 (48.90) | 18,438 (55.44) | 6.54 |
| Female | 60,006 (51.10) | 14,816 (44.56) | 6.54 |
| **Study Center** (n (%)) |  |  |  |
| Augsburg | 11,495 (9.78) | 3,223 (9.69) | 0.09 |
| Regensburg | 5,833 (4.97) | 1,285 (3.86) | 1.11 |
| Mannheim | 6,445 (5.48) | 1,862 (5.60) | 0.12 |
| Freiburg | 7,444 (6.33) | 1,223 (3.68) | 2.65 |
| Saarbrücken | 5,974 (5.09) | 1,944 (5.83) | 0.75 |
| Essen | 6,051 (5.15) | 1,939 (5.83) | 0.68 |
| Münster | 6,261 (5.33) | 1,530 (4.60) | 0.73 |
| Düsseldorf | 5,021 (4.27) | 1,606 (4.83) | 0.56 |
| Halle | 5,253 (4.47) | 2,091 (6.29) | 1.82 |
| Leipzig | 5,621 (4.78) | 2,340 (7.04) | 2.26 |
| Berlin-Nord | 6,801 (5.79) | 1,472 (4.42) | 1.37 |
| Berlin-Mitte | 7,012 (5.97) | 2,159 (6.48) | 0.51 |
| Berlin-Süd | 6,517 (5.55) | 1,373 (4.13) | 1.42 |
| Hannover | 4,697 (4.00) | 1,000 (3.01) | 0.99 |
| Hamburg | 6,544 (5.57) | 2,162 (6.50) | 0.93 |
| Bremen | 7,003 (5.96) | 1,643 (4.94) | 1.02 |
| Kiel | 5,041 (4.29) | 1,148 (3.45) | 0.84 |
| Neubrandenburg | 8,453 (7.20) | 3,254 (9.78) | 2.58 |

**Table S2: Information criteria for model with dependent variable “any care delay”**

| **Model*** | **AIC** | **BIC** |
| --- | --- | --- |
| 0 | 127116.9 | 127279.6 |
| 1 | 127111.6 | 127532.8 |
| 2 | 127120.6 | 127398.3 |
| 3 | 127123.4 | 127381.9 |
| 4 | 127113.4 | 127295.3 |
| *Independent variables for the different Models:  Model 0: age + sex + education + net equivalence household income + any cancer + any cardiovascular disease + any metabolic disease + any psychiatric disorder + east/west/berlin + urban/rural  Model 1: Model 0 + interaction east/west/berlin and age + interaction east/west/berlin and sex + interaction east/west/berlin and age and sex  Model 2: Model 0 + interaction east/west/berlin and age + interaction east/west/berlin and sex  Model 3: Model 0 + interaction east/west/berlin and age  Model 4: Model 0 + interaction east/west/berlin and sex | | |

**Table S3: Information criteria for model with dependent variable “patient-induced care delay”**

| **Model** | **AIC** | **BIC** |
| --- | --- | --- |
| 0 | 85224.93 | 85387.67 |
| 1 | 85220.7 | 85641.91 |
| 2 | 85230.82 | 85508.44 |
| 3 | 85228.75 | 85487.22 |
| 4 | 85226.73 | 85408.62 |
| *Independent variables for the different Models:  Model 0: age + sex + education + net equivalence household income + any cancer + any cardiovascular disease + any metabolic disease + any psychiatric disorder + east/west/berlin + urban/rural  Model 1: Model 0 + interaction east/west/berlin and age + interaction east/west/berlin and sex + interaction east/west/berlin and age and sex  Model 2: Model 0 + interaction east/west/berlin and age + interaction east/west/berlin and sex  Model 3: Model 0 + interaction east/west/berlin and age  Model 4: Model 0 + interaction east/west/berlin and sex | | |

**Table S4: Information criteria for model with dependent variable “provider-induced care delay”**

| **Model** | **AIC** | **BIC** |
| --- | --- | --- |
| 0 | 92827.61 | 92990.35 |
| 1 | 92803.81 | 93225.02 |
| 2 | 92825.82 | 93103.43 |
| 3 | 92824.46 | 93082.93 |
| 4 | 92828.79 | 93010.68 |
| *Independent variables for the different Models:  Model 0: age + sex + education + net equivalence household income + any cancer + any cardiovascular disease + any metabolic disease + any psychiatric disorder + east/west/berlin + urban/rural  Model 1: Model 0 + interaction east/west/berlin and age + interaction east/west/berlin and sex + interaction east/west/berlin and age and sex  Model 2: Model 0 + interaction east/west/berlin and age + interaction east/west/berlin and sex  Model 3: Model 0 + interaction east/west/berlin and age  Model 4: Model 0 + interaction east/west/berlin and sex | | |

**Table S5: Association of individual and regional characteristics and any care delay for the whole sample (n=106.191 with complete data) and for people with preexisting chronic conditions (n=83.929 with complete data)**

|  | **Whole sample** | | **Preexisting chronic conditions** | |
| --- | --- | --- | --- | --- |
|  | | | | |
| **Age** |  | |  | |
| 20-29 years | 0.95 (0.85 – 1.05) | | 0.92 (0.78 – 1.06) | |
| 30-39 years | 1.18 (1.13 – 1.24) | | 1.12 (1.06 – 1.18) | |
| 40-49 years | 1.19 (1.15 – 1.24) | | 1.17 (1.12 – 1.23) | |
| 50-59 years |  | 1.00 (Reference) | |  |
| 60-69 years | 0.80 (0.77 – 0.83) | | 0.82 (0.79 – 0.86) | |
| 70+ years | 0.59 (0.57 – 0.62) | | 0.62 (0.59 – 0.65) | |
| **Sex** |  | |  | |
| Male |  | | 1.00 (Reference) | |
| Female | 1.30 (1.27 – 1.34) | | 1.30 (1.26 – 1.34) | |
| **Education** |  | |  | |
| High |  | | 1.00 (Reference) | |
| Middle/Low | 0.97 (0.94 – 1.00) | | 0.96 (0.93 – 0.99) | |
| **Equivalized net household income** (per 1000 €) | 0.94 (0.93 – 0.95) | | 0.94 (0.93 – 0.95) | |
| **Any cancer** |  | |  | |
| No |  | | N.A. | |
| Yes | 1.15 (1.09 – 1.21) | | N.A. | |
| **Any cardiovascular disease** |  | |  | |
| No |  | | N.A. | |
| Yes | 1.20 (1.16 – 1.24) | | N.A | |
| **Any metabolic disease** |  | |  | |
| No |  | | N.A | |
| Yes | 1.20 (1.16 – 1.23) | | N.A | |
| **Any psychiatric disorder** |  | |  | |
| No |  | | N.A | |
| Yes | 1.41 (1.36 – 1.46) | | N.A | |
| **East/West/Berlin** |  | |  | |
| West |  | | 1.00 (Reference) | |
| East | 1.02 (0.98 – 1.06) | | 1.03 (0.99 – 1.08) | |
| Berlin | 1.10 (1.06 – 1.14) | | 1.10 (1.05 – 1.15) | |
| **Urban/rural** |  | |  | |
| Urban |  | | 1.00 (Reference) | |
| Rural | 1.01 (0.98 – 1.05) | | 1.00 (0.96 – 1.05) | |
